# B-Cell Receptor-Associated Protein 31 Deficiency Aggravates Ethanol-Induced Liver Steatosis and Liver Injury via Attenuating Fatty Acid Oxidation and Glycogen Synthesis

**DOI:** 10.3390/ijms262412173

**Published:** 2025-12-18

**Authors:** Shubin Yu, Yaodong Xia, Chunyan Zhang, Xiangyue Han, Xiaoyue Feng, Liya Li, Hang Ma, Jialin Xu

**Affiliations:** 1 Institute of Biochemistry and Molecular Biology, College of Life and Health Sciences, Northeastern University, Shenyang 110169, China; a1041113848@163.com (S.Y.); xiayaodong321@163.com (Y.X.); zcy9809@163.com (C.Z.); hanxiangyue928@163.com (X.H.); 17862590835@163.com (X.F.); 2 Key Laboratory of Bioresource Research and Development of Liaoning Province, College of Life and Health Sciences, Northeastern University, Shenyang 110819, China; lyli@mail.neu.edu.cn; 3 Institute of Microbial Pharmaceuticals, College of Life and Health Sciences, Northeastern University, Shenyang 110819, China; 4 Bioactive Botanical Research Laboratory, Department of Biomedical and Pharmaceutical Sciences, College of Pharmacy, The University of Rhode Island, Kingston, RI 02881, USA

**Keywords:** BAP31, Ppp1r3c, liver steatosis, fatty acid oxidation, glycogen

## Abstract

Alcoholic liver disease (ALD) is a spectrum of alcohol-induced disorders and represents a major global health challenge. B-cell receptor-associated protein 31 (BAP31) is an endoplasmic reticulum-resident chaperone involved in protein transport, apoptosis, cancer biology, and lipid metabolism. To explore its role in ALD, we used hepatocyte-specific BAP31 knockout mice (BAP31-LKO) and wild-type (WT) littermates exposed to ethanol to assess BAP31′s biochemical and metabolic impact. Following ethanol exposure, BAP31-LKO mice exhibited elevated serum alanine transaminase (23.2%, *p* < 0.05) and aspartate transaminase (31.4%, *p* < 0.05) levels compared to WT mice. Increased malondialdehyde (8.5%, *p* < 0.05) and reduced superoxide dismutase (22.8%, *p* < 0.05) in BAP31-LKO mice indicate exacerbated liver injury. Furthermore, BAP31 deficiency increased triglyceride (35.7%, *p* < 0.05) and free fatty acid (16.2%, *p* < 0.05) accumulation following ethanol treatment, while the expression of fatty acid oxidation-related genes, including Pparα, Cd36, Fatp2, Cpt2, and Acox1, was reduced in BAP31-LKO mice. The mRNA levels of Xbp1, Xbp1s, and Chop, as well as protein levels of p-eIF2α, IRE1α, GRP78, and CHOP, were increased in BAP31-LKO mice compared to WT controls, indicating aggravated ethanol-induced ER stress. Hepatic glycogen content was also reduced in BAP31-LKO mice, along with reduced Ppp1r3c expression, demonstrating impaired glycogen synthesis. Consistently, BAP31 knockdown amplified ethanol-induced lipid accumulation, inflammation, impaired glycogen storage, ER stress, and suppression of Pparα signaling in HepG2 cells. Together, these findings demonstrate that BAP31 deficiency exacerbates ethanol-induced liver steatosis, inflammation, and liver injury by impairing fatty acid oxidation and glycogen synthesis, and by amplifying ER stress responses.

## 1. Introduction

Alcohol is a globally popular beverage, but excessive drinking harms human health, contributing to liver damage and liver disease. Alcohol is primarily absorbed through the intestines and subsequently metabolized in the liver, where ethanol undergoes oxidative metabolism, converting into acetaldehyde and acetic acid [1,2]. Excessive alcohol consumption inhibits alcohol dehydrogenase (ADH), leading to the accumulation of ethanol in the liver and subsequently causing alcoholic liver disease (ALD) [3]. ALD encompasses a spectrum of progressive liver pathologies, ranging from simple steatosis to severe liver damage, including steatohepatitis, liver fibrosis, cirrhosis, and hepatocellular carcinoma [4,5]. The mechanisms underlying the progression from initial steatosis to severe ALD include lipid peroxidation, mitochondrial dysfunction, inflammatory response, oxidative stress, and alterations in innate immune regulation [6]. In the United States, more than 50% of the population consumes alcohol, and 23.1% participate in heavy drinking or binge drinking at least once per month [7]. Today, ALD has become one of the major global health issues.

Liver steatosis is the most common symptom of liver damage caused by alcohol consumption, and its severity is closely associated with the progression of ALD [8,9,10]. Liver steatosis is characterized by lipid accumulation exceeding 5% of total liver weight. Disruption of the balance between lipid synthesis and lipid catabolism is the primary cause of liver steatosis. Alcohol promotes fatty acid and triglyceride (TG) synthesis and reduces the excretion of TG export from hepatocytes, thereby disrupting the balance between TG secretion and synthesis, and ultimately leading to excessive lipid deposition and fatty liver disease [11]. Excessive alcohol consumption impairs fatty acid catabolism, primarily by inhibiting mitochondrial oxidation [12], reducing the NAD^+^/NADH ratio, and disrupting mitochondrial β-oxidation. Hepatic β-oxidation is regulated by peroxisomal acyl-coenzyme A oxidase 1 (Acox1) and carnitine palmitoyltransferase 1A (Cpt1a), direct targets of peroxisome proliferator-activated receptor α (Pparα), the master regulator of hepatic lipid metabolism [13,14,15]. Activation of fatty acid oxidation increases hepatic energy consumption and reduces lipid accumulation. Sustained activation of Pparα by endogenous ligands attenuates obesity and restores glucose homeostasis via upregulation of fatty acid oxidation-related genes [16]. Fibrates, including fenofibrate and clofibrate, are widely used clinically to treat hypertriglyceridemia and dyslipidemia, and regulate lipid and glucose metabolism by activating the PPARα signaling pathway [17]. Conversely, defects in Pparα-inducible fatty acid oxidation result in more severe hepatic steatosis during fasting, attributed to dysfunctional peroxisomal β-oxidation and loss of Acox1 activity. Similarly, alcohol exposure modulates lipid-regulating factors and lipogenic enzymes, including acetyl-CoA carboxylase (Acc), fatty acid synthase (Fas), and stearoyl coenzyme-A desaturase 1 (Scd1) [18].

As the primary site of endogenous glucose metabolism, the liver regulates glucose homeostasis through glycogenolysis and gluconeogenesis. Glycogen synthesis is essential for maintaining glucose balance and energy supply [19]. Chronic ethanol consumption disrupts glycogen synthase expression, decreases total and phosphorylated glycogen synthase protein, and leads to glycogen depletion and impaired hepatic energy metabolism [20]. Alcohol-induced fatty liver is modulated by hepatic glycogen levels. Overexpression of protein phosphatase 1 regulatory subunit 3g (Ppp1r3g) in hepatocytes increases hepatic glycogen accumulation, reduces hepatic steatosis, and mitigates alcohol-induced liver injury, suggesting a functional relationship between glycogen metabolism and ALD pathology [21].

The BAP31 gene is located on chromosomal Xq28 and encodes an endoplasmic reticulum (ER)-localized chaperone involved in protein trafficking, folding quality control, apoptosis, and ER-associated degradation [22]. Due to its key role in ER biology, BAP31 has been implicated in lipid metabolism. A contiguous deletion of ABCD1 and BAP31 results in liver dysfunction, cholestasis, hepatomegaly, and mild to moderate fibrosis [23]. Our previous study demonstrates that hepatocyte-specific deficiency of BAP31 leads to metabolic defects and liver injury. BAP31-deficient mice exhibited increased body weight and elevated hepatic lipid accumulation, driven by increased SREBP signaling and de novo lipogenesis [24]. A subsequent study using the same mouse model showed that BAP31 deficiency increased p-eIF2α, activating transcription factor 4 (ATF4) and C/EBP homologous protein (CHOP) expression and exacerbated tunicamycin-induced ER stress-associated steatosis [25]. Recently, sirtuin 2 (SIRT2) was shown to attenuate ER stress-induced hepatocyte apoptosis through BAP31 deacetylation in a chronic ALD model, further supporting a functional connection between BAP31 and ALD progression [26]. Given that ALD profoundly alters hepatic glucose and lipid metabolism, the current study aimed to investigate the biological role of BAP31 in ALD using both animal and cellular models.

## 2. Results

### 2.1. BAP31 Deficiency Aggravated Ethanol-Induced Liver Injury in Mice

**Figure 1:**
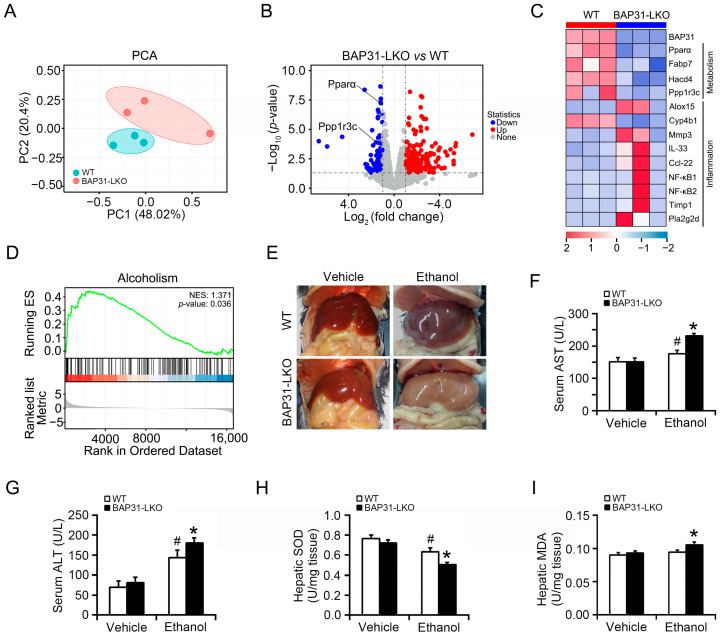
BAP31 deficiency aggravated ethanol-induced liver injury in mice. (**A**) Principal component analysis (PCA) score plot of the RNA-seq dataset of 6 liver samples from WT and BAP31-LKO mice. (**B**) Volcano plots of genes with significant differences in expression (fold change) in the livers of BAP31-LKO mice using WT mice as the benchmark. Red and blue dots indicate significantly upregulated and downregulated expressed genes (*p* < 0.05 and fold change > 1). (**C**) The hierarchical clustering heatmap of differentially expressed genes (DEGs) in the livers from WT and BAP31-LKO mice enriched in metabolism and inflammation. (**D**) Gene set enrichment analysis (GSEA) of DEGs using the GSEA Kyoto encyclopedia of genes and genomes (KEGG) pathways database (NES > 1, *p* < 0.05). (**E**) Representative photos of the livers from four groups of mice. (**F**) Serum ALT and (**G**) serum AST were determined in four groups of mice. (**H**) Hepatic SOD and (**I**) hepatic MDA were measured in four groups of mice. *n* = 8 for each group. ^#^ *p* < 0.05, compared with vehicle treatment. * *p* < 0.05, compared with WT mice.

The PCA score plot demonstrated that the samples from WT and BAP31-LKO mice were clearly separated (Figure 1A). Transcriptomic comparison between WT and BAP31-LKO mice revealed 584 differentially expressed genes (DEGs), 170 genes downregulated, and 414 genes upregulated in BAP31-LKO mice (Figure 1B). Functional enrichment analysis suggested that BAP31 deficiency regulated genes related to metabolism and inflammation. Specifically, BAP31 deficiency downregulated Pparα and Ppp1r3c, affecting fatty acid metabolism and glycogen metabolism while upregulating Nf-κB1 and Nf-κB2, suggesting enhanced inflammatory responses (Figure 1B,C). GSEA analysis further showed significant enrichment of the alcoholism signaling pathway following BAP31 loss, supporting its role in alcohol-responsive metabolic regulation (Figure 1D). To validate the transcriptomic findings, WT and BAP31-LKO mice were challenged with alcohol. The livers of BAP31-LKO mice appeared paler and greyer than those of WT mice, suggesting exacerbated fatty liver (Figure 1E). Alcohol administration significantly increased serum ALT and AST levels in both groups; however, the elevation was more pronounced in BAP31-LKO mice, indicating aggravated liver injury (Figure 1F,G). Furthermore, hepatic SOD activity was reduced in BAP31-LKO mice (Figure 1H), while MDA levels were elevated compared with WT mice (Figure 1I), demonstrating enhanced ethanol-induced oxidative stress due to BAP31 deficiency.

### 2.2. BAP31 Deficiency Promoted Ethanol-Induced Liver Steatosis in Mice

**Figure 2:**
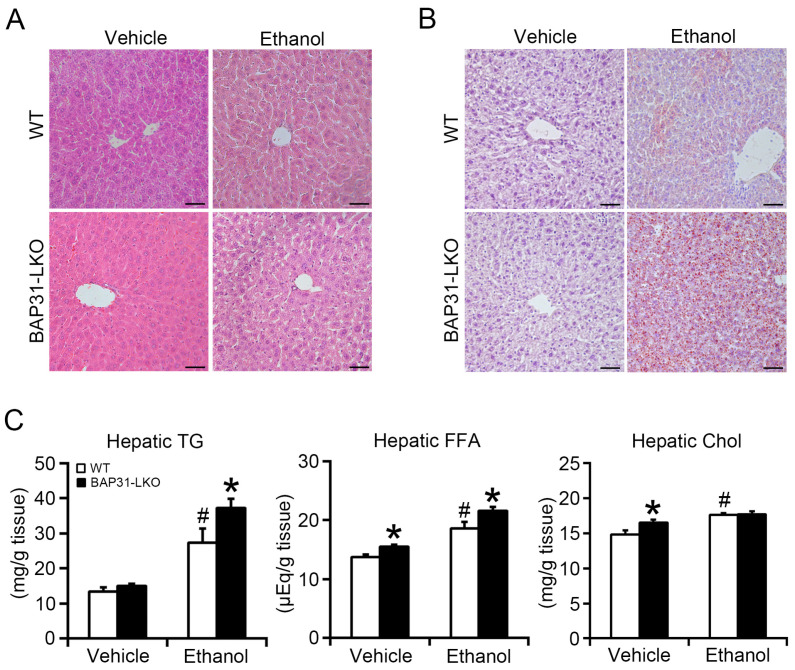
BAP31 deficiency promoted ethanol-induced liver steatosis in mice. (**A**) Representative images of hematoxylin and eosin (H/E) staining and (**B**) Oil red O staining of the livers from four groups of mice (200×, scale bar = 50 μm). *n* = 4 per group. (**C**) The lipids were purified from liver tissues of four groups of mice. The concentrations of triglycerides (TG), free fatty acid (FFA), and cholesterol (Chol) were determined using reagent kits. *n* = 8 for each group. ^#^ *p* < 0.05, compared with vehicle treatment. * *p* < 0.05, compared with WT mice.

No significant structural changes were observed in WT or BAP31-LKO mice after ethanol administration (Figure 2A). However, hematoxylin and eosin (H&E) staining and Oil red O staining revealed greater lipid accumulation in BAP31-LKO mice than in WT controls after ethanol administration (Figure 2A,B), indicating aggravated steatosis. Biochemical lipid profiling showed significantly elevated hepatic FFA and cholesterol levels in BAP31-LKO mice compared with WT controls at baseline. Ethanol also increased hepatic lipids in both genotypes, but to a greater extent in BAP31-LKO mice, as demonstrated by elevated TG (35.7%) and FFA (16.2%) levels (Figure 2C). These findings indicate that BAP31 deficiency enhances ethanol-induced lipid accumulation in the liver.

Compared with WT controls, serum glucose was reduced in BAP31-LKO mice after ethanol treatment, demonstrating more severe ethanol-induced hypoglycemia and impaired glucose regulation in the liver. Serum FFA reduced in WT mice after ethanol administration. Serum FFA levels decreased in WT mice following ethanol treatment; however, this reduction was reversed in BAP31-LKO mice, which instead exhibited a marked increase, suggesting impaired fatty acid utilization. No significant differences in serum TG, cholesterol, HDL-C, or LDL-C were observed between genotypes after ethanol treatment (Table 1).

### 2.3. BAP31 Deficiency Decreased Fatty Acid Oxidation-Related Gene Expression

**Figure 3:**
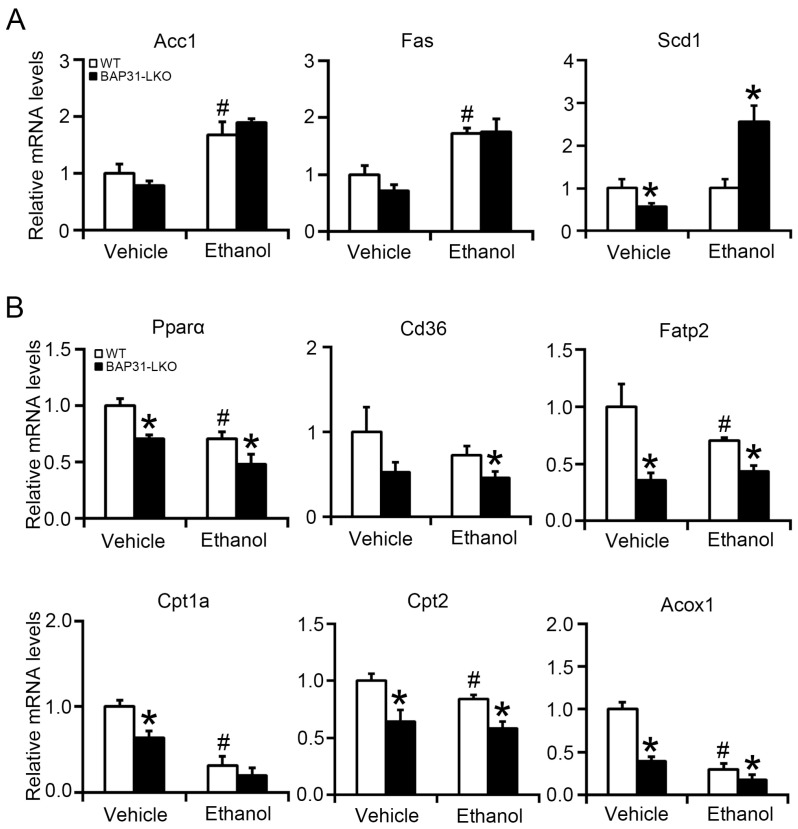
BAP31 deficiency decreased fatty acid oxidation-related gene expression. The relative mRNA levels of (**A**) Acc1, Fas, Scd1, and (**B**) Pparα, Cd36, Fatp2, Cpt1a, Cpt2, and Acox1 genes were determined by using quantitative real-time PCR analysis in the livers from four groups of mice. The relative mRNA levels have been normalized with the expression levels of the housekeeping gene 18S rRNA. *n* = 8 for each group. ^#^ *p* < 0.05, compared with vehicle treatment. * *p* < 0.05, compared with WT mice.

To explore mechanisms underlying lipid accumulation, lipogenic gene expression was evaluated by quantitative real-time PCR analysis. No significant differences in Acc1 or Fas expression were observed between groups following ethanol exposure, whereas Scd1 expression was elevated in BAP31-LKO mice (Figure 3A). Next, expression of fatty acid oxidation-related genes within the Pparα pathway was assessed. At baseline, BAP31-LKO mice exhibited significantly lower expression of Pparα, Fatp2, Cpt1a, Cpt2, and Acox1 compared with WT controls. Ethanol treatment also suppressed Pparα, Cd36, Fatp2, Cpt2, and Acox1 expressions, with reductions being more severe in BAP31-LKO mice (Figure 3B). These results suggest that BAP31 deficiency suppresses fatty acid oxidation transcriptional process, leading to reduced lipid utilization and exacerbated steatosis.

### 2.4. BAP31 Deficiency Increased Ethanol-Induced ER Stress in Mice Livers

**Figure 4:**
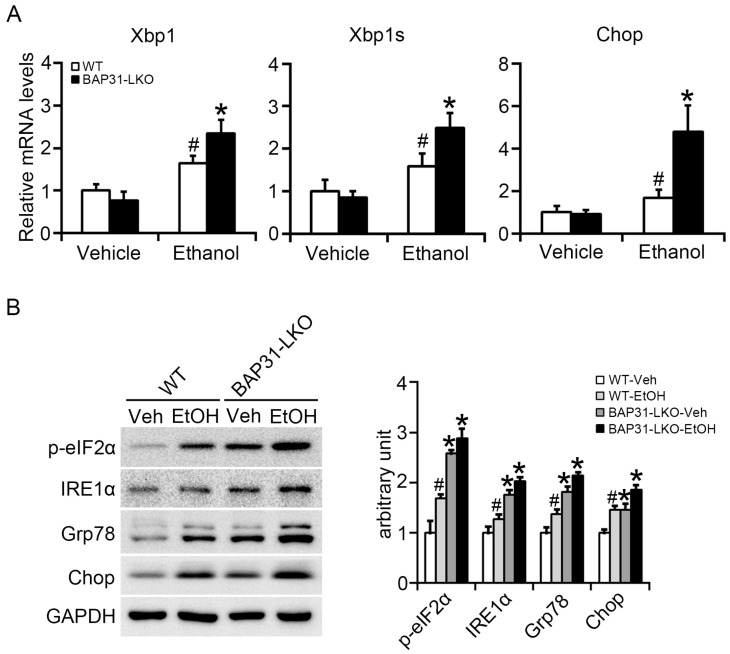
BAP31 deficiency increased ethanol-induced ER stress in mice livers. (**A**) The relative mRNA levels of ER stress related genes of Xbp1, Xbp1s, and Chop were determined by using quantitative real-time PCR analysis in the livers from four groups of mice. The relative mRNA levels have been normalized with the expression levels of the housekeeping gene 18S rRNA. *n* = 8 for each group. (**B**) The protein levels of p-eIF2α, IRE1α, Grp78, and Chop were determined by using Western blotting analysis in the livers of WT and BAP31-LKO mice treated with vehicle (Veh) or ethanol (EtOH) for 24 h. The experiment has been repeated three times independently. *n* = 3 for each group. ^#^ *p* < 0.05, compared with vehicle treatment. * *p* < 0.05, compared with WT mice.

Ethanol administration upregulated Xbp1, spliced Xbp1 (Xbp1s), and Chop mRNA expression, indicating activation of ER stress pathways [27]. These genes were expressed at significantly higher levels in BAP31-LKO mice following ethanol treatment (Figure 4A). Western blot analysis confirmed these observations by showing increased protein levels of p-eIF2α, IRE1α, GRP78, and CHOP in BAP31-LKO mice with or without ethanol exposure (Figure 4B). Together, these results indicate that BAP31 deficiency amplifies ER stress activation during ethanol challenge.

### 2.5. BAP31 Deficiency Reduced Glycogen Storage and Inhibited Glycogen Synthesis in Liver Cells

**Figure 5:**
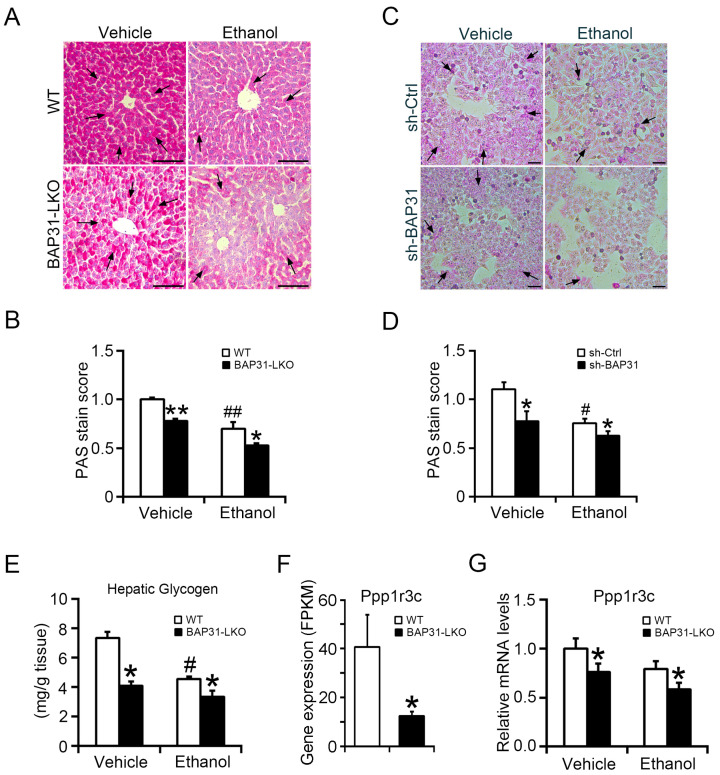
BAP31 deficiency reduced hepatic glycogen content and inhibited glycogen synthesis in liver cells. (**A**) Representative images of PAS staining in the livers from four groups of mice. Glycogen-positive area is shown in dark pink, whereas nuclei are blue. Scale bar = 100 μm. *n* = 4 per group. Black arrows indicate positive area. (**B**) PAS score analysis in panel (**A**). (**C**) sh-Ctrl and sh-BAP31 HepG2 cells were treated with vehicle and ethanol (100 mM) for 24 h. PAS staining was performed to evaluate cellular glycogen content. Scale bar = 100 μm. Data represent a representative experiment (from three independent experiments). Black arrows indicate positive area. (**D**) PAS score analysis in panel (**C**). (**E**) Hepatic glycogen was extracted. The glycogen content was determined in four groups of mice by spectrophotometry. *n* = 8 for each group. (**F**) RNA-seq-based gene expression values (FPKM) for Ppp1r3c in WT and BAP31-LKO mice. *n* = 3 for each group. (**G**) The relative mRNA level of Ppp1r3c was determined in four groups of mice by using quantitative real-time PCR analysis. The relative mRNA levels have been normalized with the expression levels of housekeeping gene 18S rRNA. *n* = 8 for each group. ^#^ *p* < 0.05, ^##^ *p* < 0.01, compared with vehicle treatment. * *p* < 0.05, ** *p* < 0.01, compared with WT mice.

Alcohol exposure disrupted glycogen synthesis, resulting in glycogen depletion and glucose disturbance [28]. Reduced PAS staining in WT mice confirmed glycogen depletion. BAP31 deficiency further reduced PAS staining under both basal and ethanol-treated conditions (Figure 5A,B). Similarly, in HepG2 cells, ethanol treatment reduced glycogen content, and this reduction was exacerbated by BAP31 knockdown, confirming its positive role in glycogen storage (Figure 5C,D). Direct biochemical quantification showed reduced hepatic glycogen content in BAP31-LKO mice under vehicle conditions, with further reduction after ethanol treatment (Figure 5E). Transcriptomic analysis revealed decreased Ppp1r3c expression in BAP31-LKO mice (Figure 5F), which was confirmed by qPCR. Ethanol exposure further suppressed Ppp1r3c, with significantly lower expression in BAP31-LKO mice (Figure 5G). These findings indicate that BAP31 deficiency impairs glycogen synthesis via downregulation of Ppp1r3c, contributing to reduced liver glycogen storage.

### 2.6. BAP31 Deficiency Increased Ethanol-Induced Lipid Accumulation, ER Stress and Inflammatory Response in HepG2 Cells

**Figure 6:**
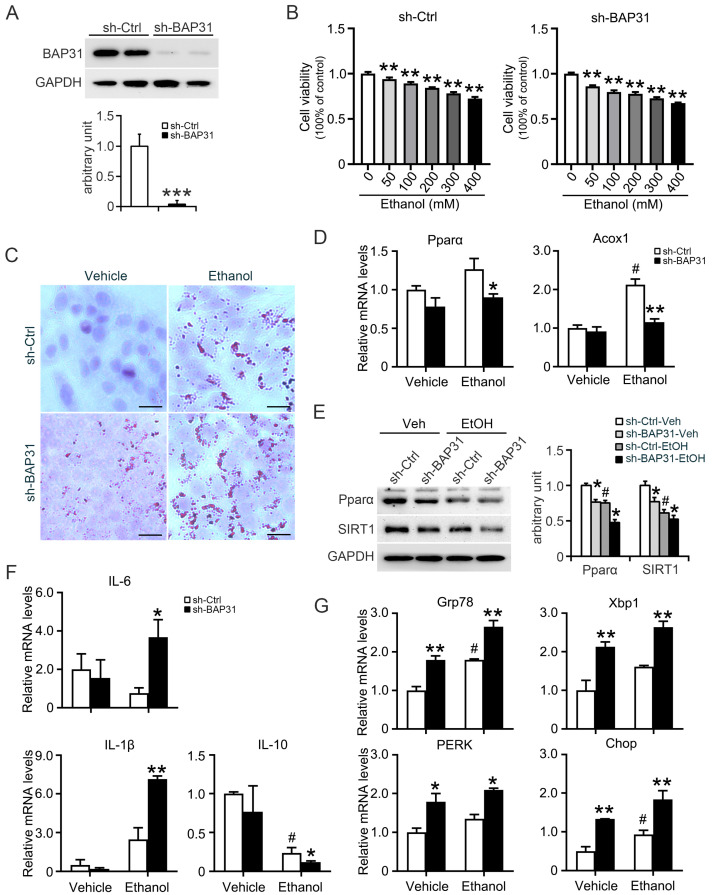
BAP31 deficiency increased ethanol-induced lipid accumulation, ER stress and inflammatory response in HepG2 cells. (**A**) BAP31 protein levels were determined by using Western blotting analysis in sh-Ctrl and sh-BAP31 HepG2 cells, *** *p* < 0.001, compared with sh-Ctrl HepG2 cells. (**B**) Cell viability was evaluated in sh-Ctrl (**left panel**) and sh-BAP31 (**right panel**) HepG2 cells treated with various concentrations of ethanol (0, 50, 100, 200, 300, 400 mM) for 24 h. ** *p* < 0.01, compared with vehicle treatment. (**C**) Lipid accumulation was determined via Oil red O staining in sh-Ctrl and sh-BAP31 HepG2 cells treated with vehicle and ethanol (100 mM) for 24 h. Scale bar = 25 μm. (**D**) The transcriptional levels of Pparα and Acox1 were determined in sh-Ctrl and sh-BAP31 HepG2 cells treated with vehicle and ethanol (100 mM) for 24 h. ^#^ *p* < 0.05, compared with vehicle treatment. * *p* < 0.05, ** *p* < 0.01, compared with sh-Ctrl HepG2 cells. (**E**) The protein levels of Pparα and SIRT1 were determined in sh-Ctrl and sh-BAP31 HepG2 cells treated with vehicle and ethanol (100 mM) for 24 h. ^#^ *p* < 0.05, compared with vehicle treatment. * *p* < 0.05, compared with sh-Ctrl HepG2 cells. (**F**) The transcriptional levels of IL-6, IL-1β and IL-10 were determined in sh-Ctrl and sh-BAP31 HepG2 cells treated with vehicle and ethanol (100 mM) for 24 h.^#^ *p* < 0.05, compared with vehicle treatment. * *p* < 0.05, ** *p* < 0.01, compared with sh-Ctrl HepG2 cells. (**G**) The transcriptional levels of Grp78, Xbp1, PERK and Chop were determined in sh-Ctrl and sh-BAP31 HepG2 cells treated with vehicle and ethanol (100 mM) for 24 h. ^#^ *p* < 0.05, compared with vehicle treatment. * *p* < 0.05, ** *p* < 0.01, compared with sh-Ctrl HepG2 cells.

To further validate these observations, in vitro assays were conducted. BAP31 knockdown was confirmed in HepG2 cells (Figure 6A). Ethanol reduced cell viability in a concentration-dependent manner in both cell types, and a concentration of 100 mM was selected for subsequent assays (Figure 6B). Oil Red O staining showed increased lipid accumulation in sh-Ctrl cells following ethanol treatment, with even higher lipid levels in sh-BAP31 cells (Figure 6C).

The results from in vivo model were supported by cell-based assays. In HepG2 cells, BAP31 knockdown reduced expression of Pparα and Acox1 at both mRNA and protein levels (Figure 6D,E). The expression of inflammatory markers IL-6 and IL-1β was significantly increased, while anti-inflammatory IL-10 was reduced in BAP31-deficient cells following ethanol exposure (Figure 6F). Additionally, ER stress-associated genes (Grp78, Xbp1, PERK, and Chop) were markedly increased in sh-BAP31 cells with or without ethanol (Figure 6G). Together, these findings demonstrate that BAP31 deficiency promotes ethanol-induced lipid accumulation, inflammation, and ER stress while impairing fatty acid oxidation signaling.

## 3. Discussion

The current study showed the function of BAP31 in regulating ethanol-induced liver steatosis and ALD, revealing its important roles in modulating lipid metabolism and fatty acid oxidation in mice. BAP31 deficiency reduced fatty acid oxidation and exacerbated liver steatosis in response to ethanol administration, primarily manifested by enhanced inflammatory responses, intensified ER stress, and depletion of hepatic glycogen reserves.

The primary characteristic of ALD is fatty liver, manifested as increased lipid accumulation within hepatocytes [29]. In this study, BAP31 deficiency exacerbated lipid accumulation in mouse hepatocytes and accelerated the progression of ethanol-induced fatty liver disease. Alcohol disturbs multiple pathways of hepatic lipid metabolism, including impaired hepatic fatty acid uptake and oxidation, increased de novo lipid synthesis, enhanced neutral lipid storage, and suppression of lipid export and lipolysis. Alcohol exposure modulates numerous lipid-regulating transcription factors and lipogenic enzymes, thereby enhancing hepatic TG accumulation [9]. SREBP-1c regulates the expression of Acc, Fas, and Scd1 [30] and is implicated in the pathogenesis of alcohol-induced steatosis. Consistent with this, SREBP-1c null mice are protected from alcohol-induced hepatic steatosis [31]. In our study, although Acc1 and Fas transcript levels remained unchanged, Scd1 expression was significantly increased in BAP31-LKO mice following ethanol treatment, suggesting enhanced monounsaturated fatty acid production that promotes TG synthesis and lipid storage.

Ethanol also inhibits hepatic fatty acid oxidation through suppression of the Pparα signaling pathway, a key nuclear receptor controlling the transcription of genes involved in fatty acid transport and β-oxidation [12]. FFAs are metabolized via mitochondrial and peroxisomal β-oxidation pathways, which are partially regulated by Cpt1 and Acox1 enzymes [32,33]. Both enzymes are direct transcriptional targets of Pparα, making this pathway essential for maintaining hepatic lipid homeostasis [34]. Pparα signaling also plays a protective role in alcohol detoxification. For instance, Pparα-null mice display more severe liver inflammation, fibrosis, oxidative stress, and hepatocyte apoptosis following ethanol administration [35]. Pharmacological Pparα agonists, such as WY-14,643 and pemafibrate, can reverse alcoholic fatty liver and improve liver function in mice and rats [36,37]. Likewise, activation of the Pparα-catalase pathway restored NAD availability and accelerated ethanol clearance, further demonstrating its importance in ALD progression [38]. In this study, BAP31 deficiency reduced the expression of Pparα and downregulated the transcriptional levels of Cpt1a, Cpt2, and Acox1 in mice livers. Also, BAP31 deficiency decreased both the mRNA and protein levels of Pparα and/or Acox1 in HepG2 cells. These findings demonstrated that BAP31 deficiency exacerbated ethanol-induced hepatic steatosis by inhibiting fatty acid oxidation. Our previous study reported that BAP31 deficiency in adipocytes impaired lipolysis and promoted abnormal growth of lipid droplets in white adipose tissue [39]. Given that fatty acids serve as natural ligands for Pparα activation [40], lipolytic flux in BAP31-deficient models may further attenuate Pparα signaling and worsen hepatic steatosis. Decreased fatty acid content in BAP31-LKO mice reduced Pparα signaling activation and decreased fatty acid oxidation (Table 1), leading to the reduction in lipid utilization in the liver, thus accelerating the progression of alcoholic fatty liver. Ethanol exposure not only increases the liver’s uptake of exogenous fatty acids and incorporates them into hepatic TG or total lipids, but also upregulates hepatic fatty acid transporters, thereby promoting fatty acid uptake and leading to excessive fat accumulation [41]. Notably, in the current study, the decreased expression of fatty acid transport genes (Cd36 and Fatp2) observed in ethanol-treated BAP31-LKO mice suggests that increased lipid accumulation is not driven by enhanced fatty acid uptake, but rather by impaired utilization and oxidation.

In addition to lipid dysregulation, ethanol disrupted hepatic glucose metabolism. Acute ethanol exposure reduces Akt phosphorylation, suppressing downstream GSK3β inhibition and subsequently reducing glycogen synthase activity and glycogen storage [42]. On the contrary, pharmaceutical activation of Akt alleviated ethanol-induced fatty liver in mice [43], highlighting the metabolic link between insulin signaling and ALD. Glycogen synthesis is mediated by protein phosphatase 1 (PP1) regulatory subunits, including the liver-enriched isoforms Ppp1r3a–Ppp1r3g. Previous studies showed that hepatocyte-specific overexpression of Ppp1r3g restored glycogen accumulation and attenuated ethanol-induced liver injury and steatosis [20]. Depletion of Ppp1r3c significantly reduced intracellular glycogen levels in mouse chondrocytes and suppressed the neoplastic phenotype [44]. Similarly, Akt directly phosphorylates Ppp1r3g, and loss of Ppp1r3g markedly impairs insulin responsiveness. Conversely, restoring Ppp1r3g expression significantly increases hepatic glycogen storage, improves glucose clearance, and enhances insulin sensitivity, establishing an insulin/Akt/Ppp1r3g regulatory axis that governs glycogen synthesis and glucose homeostasis [45]. Multiple mechanisms regulate Ppp1r3c expression as well. For example, hypoxia-inducible factor-1 (HIF-1) activates Ppp1r3c transcription by binding to a functional hypoxia-response element located 229 bp upstream of its promoter, thereby promoting glycogen accumulation as an adaptive response to hypoxia in human MCF-7 cells [46]. Consistent with this, we observed significantly reduced hepatic glycogen and decreased Ppp1r3c expression in BAP31-LKO mice. Together, these data suggest that BAP31 is required for maintaining hepatic glycogen homeostasis, and its deficiency exacerbates ethanol-induced dysregulation of glucose metabolism.

ER stress is another hallmark of ALD progression [47]. Ethanol metabolism increases oxidative and metabolic burden on the ER, triggering the unfolded protein response (UPR) and activation of PERK/eIF2α, ATF6, and IRE1/XBP1 signaling pathways [48,49]. Persistent ER stress promotes several key drivers of ALD, such as lipogenesis, inflammation, and hepatocyte apoptosis. In this study, BAP31 deficiency markedly increased ER stress markers (GRP78, XBP1, CHOP, and p-eIF2α) at both transcriptional and protein levels, consistent with prior studies showing that BAP31 modulates ER-mitochondria signaling, caspase-8-mediated apoptosis, and mitochondrial respiratory activity [25,50,51,52].

Heavy alcohol consumption also triggers immune responses and hepatic inflammation through cytokine secretion and innate immune activation [53]. Following ethanol administration, BAP31-LKO mice exhibited significantly elevated serum ALT and AST levels compared with WT controls, indicating worsened hepatocellular damage. In vitro, BAP31 knockdown increased pro-inflammatory cytokines (IL-6 and IL-1β) and reduced the anti-inflammatory cytokine IL-10, further supporting a role for BAP31 in modulating inflammatory signaling. Oxidative stress is a major contributor to ALD progression [54], and consistent with this, BAP31-LKO mice showed reduced SOD activity and elevated MDA levels following ethanol treatment, reflecting increased lipid peroxidation and oxidative injury.

Together, these findings indicate that BAP31 plays a protective role in maintaining hepatic metabolic homeostasis. Its deficiency impairs fatty acid oxidation, reduces glycogen synthesis, enhances ER stress, and promotes inflammatory and oxidative responses, collectively accelerating ethanol-induced liver injury and steatosis.

## 4. Materials and Methods

### 4.1. Animal Experiment

Mice with a targeted deficiency of the BAP31 gene specifically in hepatocytes were generated as previously described [24] and were housed individually under a temperature (22–25 °C) and humidity-controlled (30–70%) animal facility, with a 12/12 h lighting schedule. Based on the experimental workflow and breeding requirements, 32 mice were ultimately selected for use. Similarly, our approach complies with the “3Rs” principle [55,56,57]. Male BAP31 conditional knockout mice (BAP31-LKO) (8-week-old, at 8 weeks of age, the organs of the mice have fully developed, their health indicators are favorable, and they are in excellent overall health [58]) and male wild type littermates (WT) (8-week-old) were divided into four groups randomly: WT-Vehicle, BAP31-LKO-Vehicle, WT-Ethanol, and BAP31-LKO-Ethanol (*n* = 8 for each group, the commonly used sample size of 8 animals per group can be statistically justified when a large to very large effect size is expected [59]). After a 6 h fast, animals were administered 25% (*w*/*v*) ethanol at a cumulative dosage of 6 g/kg body weight via four equally divided gavages at 20 min intervals. The experimental methods were referenced from some of the previous literature [60,61,62]. Calculate the gavage dose based on each mouse’s body weight. Control mice received an equal volume of isocaloric maltose solution. Twenty-four hours after the final dose, mice were anesthetized by 3% isoflurane, and blood samples were collected and centrifuged (4000× *g*, 4 °C and 30 min) for serum isolation. The livers were weighed and cut into small pieces, then stored at −80 °C. The animal protocol was approved by the Northeastern University Animal Care and Use Committee (NEU-EC-2024A026S). This study was reported following the ARRIVE 2.0 Essential 10 guidelines [63].

### 4.2. Measurement of Serum Metabolites and Liver Extracts

Serum glucose (F006-1-1), triglycerides (A110-2-1), cholesterol (Chol, F002-1-1), high-density lipoprotein cholesterol (HDL-C, A112-2-1), low-density lipoprotein cholesterol (LDL-C, A113-2-1), aspartate transaminase (AST, C010-1-1), and alanine transaminase (ALT, C009-1-1) levels were determined with the reagent kits from Jiancheng Biomedical Company (Nanjing, China). Serum free fatty acids (FFA, 294-63601) were quantified using a reagent kit from Wako Pure Chemical Industries, Ltd. (Osaka, Japan). Liver tissues (c.a. 20 mg) were homogenized following the manufacturer’s instructions. Hepatic malondialdehyde (MDA, S0131S), superoxide dismutase (SOD, S0103), and glycogen (A043-1-1) contents were analyzed with the reagent kits from Beyotime Biotechnology (Shanghai, China) and Jiancheng Biomedical Company.

### 4.3. Measurement of Hepatic Lipids

Liver tissues (~50 mg) were homogenized with 1 mL of phosphate-buffered saline, then 200 μL of tissue lysates were taken, and lipids were extracted using the mixture of chloroform-methanol (2:1; *v*/*v*) as previously described [64]. Lipid residue was dissolved in 1% Triton X-100 in 100% ethanol. Lipids were quantified as the above methods, and the relative content was normalized with tissue weight.

### 4.4. Hematoxylin and Eosin Staining, Periodic Acid-Schiff Staining, and Oil Red O Staining

1 cm × 1 cm tissue blocks were isolated from the liver tissue. Four mice were selected for each group for the experiment. Sections (5 μm) of paraffin-embedded liver were cut and stained with hematoxylin-eosin (H/E) or periodic acid-schiff (PAS) staining (Beyotime Biotechnology. C0142S) following normal procedures, mounted with glycerol-gelatin; for Oil red O staining, frozen sections or HepG2 cells (sh-Ctrl and sh-BAP31 HepG2 cells) were fixed in 4% neutral-buffered formalin for 10 min, then stained with Oil Red O solution (six parts of Oil red O stock solution and four parts of ddH_2_O; Oil red O stock solution is 0.5% Oil red O in 100% isopropanol) for 15 min, and then counterstained with hematoxylin and mounted in glycerol-gelatin; for PAS staining, HepG2 cells were fixed in 4% neutral-buffered formalin for 10 min, then stained with PAS solution for 15 min, and then counterstained with hematoxylin and mounted in glycerol-gelatin, subsequently observed using a LEICA DMI3000 B inverted microscope before histopathology analysis (Leica Biosystems; Wetzlar, Germany). For the PAS staining results, perform grayscale analysis on the raw images using ImageJ 2 (2.3.0), and analyze the grayscale data using GraphPad Prism 8.0.2.

### 4.5. Cell Culture

For the cell viability assay, sh-Ctrl and sh-BAP31 HepG2 cells were seeded in a 96-well plate with a density of 10,000 cells per well. After the cells were treated with various concentrations (0 mM, 50 mM, 100 mM, 200 mM, 300 mM, and 400 mM) of ethanol for 24 h described as before [65,66,67], 10 μL of the CCK8 solution was added to each well. The plate was subsequently incubated in the incubator for 4 h, then the absorbance at 450 nm was measured using a BioTek microplate reader (BioTek Instruments, Inc.; Winooski, VT, USA) to quantify cell viability. For gene expression quantification, sh-Ctrl and sh-BAP31 HepG2 cells were treated with vehicle (without ethanol) or ethanol (100 mM) for 24 h, and then the transcriptional and protein levels of target genes were determined by using quantitative real-time PCR or immunoblotting assay.

### 4.6. RNA Isolation and Real-Time PCR

Total RNA was isolated from 30 mg liver tissues using TRIzol reagent (Thermo Fisher Scientific Inc.; Waltham, MA, USA) according to the manufacturer’s instructions. RNA concentration and quality were assessed using Nanodrop Microvolume Spectrophotometers and gel electrophoresis. Two micrograms of total RNA were converted to single-stranded cDNA (A5003, Promega Corporation; Madison, WI, USA). The relative mRNA levels were quantified using a CFX96 Touch™ Real-Time PCR Detection System (Bio-Rad Laboratories; Hercules, CA, USA). SYBR green master mix was used (B21203, Selleck Chemicals; Houston, TX, USA). The sequences of the related primers are listed in Supplementary Table S1.

### 4.7. Transcriptome Sequencing

We selected three mice each from the WT-Vehicle group and the BAP31-LKO-Vehicle group, excised liver tissue, and performed transcriptome sequencing. Our sample size meets the minimum requirement for three biological replicates. And the economic cost is another issue that we considered. Total RNA was extracted from the 30 mg liver tissues of WT and BAP31-LKO mice using TRIzol reagent. The cDNA libraries were constructed from 2 μg of total RNA and sequenced using an Illumina-HiSeq2000/2500 platform at BGI Genomics Co., Ltd. (Shenzhen, China). FPKM values were calculated using standardized read counts based on gene length and total aligned reads. Differentially expressed genes (DEGs) were identified with a threshold of *p* < 0.05 and fold change > 1.0. Gene Set Enrichment Analysis (GSEA) was performed using the R (4.4.2).

### 4.8. Immunoblotting Analysis

Liver tissues (~30 mg) were homogenized with 1 mL of RIPA buffer, then centrifuged at 12,000× *g* for 15 min. The supernatants were collected, and the protein concentrations were measured by using the BCA assay (Thermo Fisher Scientific). Homogenates (40–80 μg) were resolved by SDS-PAGE and then transferred to a PVDF membrane. The membranes were blocked with 5% non-fat dry milk or 5% BSA for 1 h, followed by incubation with primary antibodies overnight at 4 °C, then incubated with species-appropriate HRP-conjugated secondary antibody for 1 h at room temperature. Bands were visualized with Bio-Rad ChemiDoc™ Imaging Systems (Bio-Rad Laboratories) using an ECL detection kit (Beyotime Biotechnology). Antibodies against glucose-regulated protein 78 (GRP78, #3183), CHOP (#5554), p-eIF2α (#3398), inositol-requiring enzyme 1α (IRE1α, #3294), and GAPDH (#5174) were ordered from Cell Signaling Technology (Danvers, MA, USA). Antibodies against BAP31 (11200-1-AP) and Pparα (66826-1-IG) were ordered from Proteintech (Wuhan, China), and Acox1 (A8091) from ABclonal (Wuhan, China).

### 4.9. Statistical Analysis

Quantitative data were presented as mean ± SE. Statistical differences were determined by one-way ANOVA and two-way ANOVA followed by a Duncan’s Multiple Range post hoc test using the SPSS 13.0 software (SPSS, Chicago, IL, USA). All statistical tests with *p* < 0.05 were considered significant. All experiments were conducted with three biological replicates to minimize the impact of random error, enhance the statistical significance of the results, and ensure the reliability and statistical validity of the data [55,56].

## 5. Conclusions

In summary, this study demonstrates that BAP31 plays a critical protective role against ethanol-induced liver injury. Loss of BAP31 impaired hepatic metabolic homeostasis by suppressing PPARα signaling, reducing fatty acid oxidation, and disrupting glycogen synthesis, leading to excessive lipid accumulation and exacerbated steatosis. BAP31 deficiency also intensified endoplasmic reticulum stress, oxidative damage, and inflammatory responses, further worsening alcohol-induced liver pathology. These findings identify BAP31 as an important regulator of hepatic lipid and glucose metabolism and suggest that targeting BAP31-associated pathways may offer new therapeutic opportunities for the prevention and treatment of alcoholic liver disease.

## Figures and Tables

**Table 1:** Serum parameters of wild type (WT) and BAP31-LKO mice upon ethanol or maltose solution administration for 24 h.

		Control		Ethanol	
	Unit	WT	BAP31-LKO	WT	BAP31-LKO
Glucose	mg/dL	48.71 ± 5.34	49.79 ± 3.63	68.00 ± 4.88	52.75 ± 4.47 *
TG	mg/dL	44.42 ± 5.44	41.06 ± 2.45	53.77 ± 5.11	47.94 ± 5.49
FFA	mEq/L	0.45 ± 0.06	0.36 ± 0.05	0.26 ± 0.04	0.50 ± 0.06 *
Cholesterol	mg/dL	100.21 ± 3.17	92.69 ± 3.63	84.31 ± 3.98	87.47 ± 5.83
HDL-C	mg/dL	41.98 ± 2.05	38.92 ± 1.65	32.98 ± 2.25	31.17 ± 2.92
LDL-C	mg/dL	50.82 ± 2.24	46.96 ± 3.18	43.65 ± 6.41	37.48 ± 2.57

Note: The blood was extracted and serum was isolated. Serum glucose, triglycerides (TG), free fatty acid (FFA), cholesterol (Chol), high-density lipoprotein cholesterol (HDL-C), low-density lipoprotein cholesterol (LDL-C) were determined by using reagent kits. *n* = 8 for each group. * *p* < 0.05, compared with WT mice.

## Data Availability

The original contributions presented in this study are included in the article/Supplementary Material. Further inquiries can be directed to the corresponding author.

## Supplementary Materials

The following supporting information can be downloaded at: https://www.mdpi.com/article/10.3390/ijms262412173/s1.

## Abbreviations

The following abbreviations are used in this manuscript:

ABCD1	ATP Binding Cassette Subfamily D Member 1
ACC	Acetyl-CoA Carboxylase
ACOX1	Peroxisomal Acyl-coenzyme A Oxidase 1
ADH	Alcohol Dehydrogenase
AKT	Protein Kinase B
ALD	Alcoholic Liver Disease
ALT	Alanine Aminotransferase
AST	Aspartate Aminotransferase
ATF4	Activating Transcription Factor 4
ATF6	Activating Transcription Factor 6
BAP31	B-cell Receptor-Associated Protein 31
BCL-2	B-cell Lymphoma-2
BCL-XL	B-cell Lymphoma-extra Large
CD36	Cluster of Differentiation 36
CHOL	Cholesterol
CHOP	C/EBP Homologous Protein
CPT1α	Carnitine Palmitoyltransferase 1 Alpha
CPT2	Carnitine Palmitoyltransferase 2
EIF2α	Eukaryotic Translation Initiation Factor 2 Alpha
ER	Endoplasmic Reticulum
FAS	Fatty Acid Synthase
FATP2	Fatty Acid Transport Protein 2
FFA	Free Fatty Acid
FPKM	Fragments per Kilobase of Transcript per Million Mapped Reads
GRP78	Glucose Regulated Protein 78
GSK3β	Glycogen Synthase Kinase 3 Beta
HDL-C	High-Density Lipoprotein Cholesterol
IL-10	Interleukin-10
IL-1β	Interleukin-1β
IL-6	Interleukin-6
IRE1α	Inositol Requiring Kinase Enzyme 1 Alpha
LDL-C	Low-Density Lipoprotein Cholesterol
MDA	Malondialdehyde
NF-κB1	Nuclear Factor Kappa B Subunit 1
NF-κB2	Nuclear Factor Kappa B Subunit 2
PERK	Protein Kinase RNA–like Endoplasmic Reticulum Kinase
PPARα	Peroxisome Proliferator-Activated Receptor Alpha
PPP1R3A	Protein Phosphatase 1 Regulatory Subunit 3A
PPP1R3C	Protein Phosphatase 1 Regulatory Subunit 3C
PPP1R3G	Protein Phosphatase 1 Regulatory Subunit 3G
SCD1	Stearoyl Coenzyme A Desaturase 1
SIRT1	Silent Information Regulator 1
SIRT2	Silent Information Regulator 2
SOD	Superoxide Dismutase
SREBP1C	Sterol Regulatory Element Binding Protein-1c
TG	Triglyceride
UPR	Unfolded Protein Response
XBP1	X-Box Binding Protein 1
XBP1S	Spliced X-Box Binding Protein 1
