# Supplementary material for: B-Cell Receptor-Associated Protein 31 Deficiency Aggravates Ethanol-Induced Liver Steatosis and Liver Injury via Attenuating Fatty Acid Oxidation and Glycogen Synthesis
Source: Int J Mol Sci. 2025 Dec 18;26(24):12173. doi: 10.3390/ijms262412173 (PMC12734246; doi:10.3390/ijms262412173)
Supplement: Supplementary file 1 [file ijms-26-12173-s001.zip › ijms-4004596-supplementary.pdf]

Table S1. Primer sequences for real-time PCR analysis.

Genes （Mouse）	Forward (5′ ⟶ 3′)	Reverse (5′⟶3′)
18S	AGTCCCTGCCCTTTGTACACA	CGATCCGAGGGCCTCACTA
Acc1	GATGAACCATCTCCGTTGGC	GACCCAATTATGAATCGGGAGTG
Acox1	TAACTTCCTCACTCGAAGCCA	AGTTCCATGACCCATCTCTGTC
Cd36	CGCTTTCTGCGTATCGTCTG	GATGCACGGGATCGTGTCT
Cpt1a	CTCCGCCTGAGCCATGAAG	CACCAGTGATGATGCCATTCT
Fatp2	TCCTCCAAGATGTGCGGTACT	TAGGTGAGCGTCTCGTCTCG
Fas	GGAGGTGGTGATAGCCGGTAT	TGGGTAATCCATAGAGCCCAG
Pparα	AGAGCCCCATCTGTCCTCTC	ACTGGTAGTCTGCAAAACCAAA
Scd1	TTCTTGCGATACACTCTGGTGC	CGGGATTGAATGTTCTTGTCGT
Chop	ACCTTCACTACTCTTGACCCTG	GATGTGCGTGTGACCTCTGT
Xbp1	AGCAGCAAGTGGTGGATTTG	GAGTTTTCTCCCGTAAAAGCTGA
Xbp1s	GAGTCCGCAGCAGGTG	GTGTCAGAGTCCATGGGA
Cpt2	CAGCACAGCATCGTACCCA	TCCCAATGCCGTTCTCAAAAT
Ppp1r3c	TGATCCATGTGCTAGATCCACG	ACTCTGCGATTTGGCTTCCTG

Genes (Human)	Forward (5′ ⟶ 3′)	Reverse (5′⟶3′)
18S	AGTCCCTGCCCTTTGTACACA	CGATCCGAGGGCCTCACTA
Acox1	CACAAGTAAACCAGCGTGTAAA	GTTCTTAGCCCACTCAAACAAG
Bip	GGTATTGAAACTGTGGGAGGTGTC	GATGATTGTCTTTTGTCAGGGGTCT
Chop	AGCTGGAAGCCTGGTATGAG	GTGACCTCTGCTGGTTCTGG
IL-1β	AACCTCTTCGAGGCACAAGG	GGCGAGCTCAGGTACTTCTG
IL-6	CGCTAGCCTCAATGACGACC	GGGTGGGGCTGATTGGAAAC
IL-10	TCAAGGCGCATGTGAACTCC	GATGTCAAACTCACTCATGGCT
PERK	ATCCCCCATGGAACGACCTG	ACCCGCCAGGGACAAAAATG
Pparα	CACGGAAAGCCCACTCTGC	CGATCCGAGGGCCTCACTA
Xbp1	AGTGGCCGGGTCTGCTGAGT	CAAGTTGTCCAGAATGCCCA
